# Towards the High Phase Purity of Nanostructured Alluaudite-Type Glass-Ceramics Cathode Materials for Sodium Ion Batteries

**DOI:** 10.3390/ma14174997

**Published:** 2021-09-01

**Authors:** Maciej Nowagiel, Mateusz J. Samsel, Tomasz K. Pietrzak

**Affiliations:** Faculty of Physics, Warsaw University of Technology, Koszykowa 75, PL-00-662 Warsaw, Poland; maciej.nowagiel.stud@pw.edu.pl (M.N.); mateusz.samsel.stud@pw.edu.pl (M.J.S.)

**Keywords:** glass-ceramics, nanomaterials, cathode materials, nanocrystallization, alluaudite

## Abstract

Alluaudite-type materials are systematically attracting more attention as prospective cathode materials for sodium ion batteries. In this paper, we strove to optimize various synthesis parameters of three alluaudite compositions (Na${}_{2}$Fe${}_{3}$(PO${}_{4}$)${}_{3}$—FFF, Na${}_{2}$VFe${}_{2}$(PO${}_{4}$)${}_{3}$—VFF, and Na${}_{2}$VFeMn(PO${}_{4}$)${}_{3}$—VFM) to obtain nanostructured alluaudite-type glass-ceramics with high phase purity. We systematically investigated the role of the chemical reactions, temperature and time of melting, cooling rate, and reducing factors on the quality of the final products. A detailed synthesis protocol along with X-ray diffractometry, thermal analysis, scanning electron microscopy imaging, and electrical conductivity measurements (with impedance spectroscopy) are reported. As a result, a significant increase of the conductivity was observed in the nanomaterials. The highest value was reached for the VFF composition and was equal to $6\times 10^{-4}$ S/cm at room temperature.

## 1. Introduction

The rapidly increasing development of and global demand for portable electronics, electric vehicles, and stationary storage for renewable energy sources encourage looking beyond well-established lithium ion battery technologies. Therefore, it comes as no surprise that the interest in Na${}^{+}$ ion conductors has recently revived, driven by a vision of sodium ion batteries (NIBs). Due to the common abundance of sodium, NIBs (despite their slightly lower OCV and capacity) could be a solution to shrinking resources of lithium. Therefore, many candidates for NIB chemistry have been studied. Among them, NASICON-type materials have drawn much interest, with the possibility to be used as solid electrolytes (predominant ionic conductors) and electrode materials (mixed electronic ionic conductors). On the contrary, alluaudite-type materials (e.g., ${\mathrm{NaMnFe}}_{2}{\left({\mathrm{PO}}_{4}\right)}_{3}$) are much less known in this field, even though their ability to reversibly intercalate sodium ions was demonstrated almost 20 years ago.

Alluaudites were firstly discovered in natural minerals. In 1848, Damour studied iron, manganese, and sodium phosphate derived from the pegmatite of Chanteloube near Limoges in central France. He postulated classifying this mineral into separate species. He also proposed naming it after a French mineralogist François Alluaud [1]. It was not until 1955 that Fisher described the structure of alluaudite. It belongs to the monoclinic system and the C2/c space group. The unit cell parameters of a material containing iron and manganese in a proportion of $\frac{\mathrm{Fe}}{\mathrm{Mn}}=1.76$ were determined as follows: a=12.004Å, b=12.533Å, c=6.404Å, $\beta =11422^{\prime }$ [2].

Sixteen years later, Moore made a detailed structural analysis. The ideal crystalline compounds with an alluaudite structure can be described by a number of chemical formulas, limited, on the one hand, by the composition, in which there are lower (2+) oxidation states of manganese and iron—${\mathrm{NaCaMn}}^{2+}{\mathrm{Fe}}_{2}^{2+}{\left({\mathrm{PO}}_{4}\right)}_{3}$—and on the other hand, in which these metals are at higher (3+) valence state, which at the same time results in the absence of sodium and calcium in the composition—${\mathrm{Mn}}^{3+}{\mathrm{Fe}}_{2}^{3+}{\left({\mathrm{PO}}_{4}\right)}_{3}$. Alluaudites tend to crystallize in a partially oxidized form with a chemical composition somewhere in between. Alluaudites are characterized by the simultaneous presence of iron at different degrees of oxidation (2+,3+), which can be expressed by a formula: ${\mathrm{Na}}_{2}{\mathrm{Mn}}^{2+}\left({\mathrm{Fe}}^{2+}{\mathrm{Fe}}^{3+}\right){\left({\mathrm{PO}}_{4}\right)}_{3}$. In general, compounds with an alluaudite structure can be described by a structural formula $4X\left(1\right)X\left(2\right)M\left(1\right)M{\left(2\right)}_{2}{\left({\mathrm{TO}}_{4}\right)}_{3}$, where X is a large cation with a polyhedral environment of oxygen ions (e.g., ${\mathrm{Na}}^{+},{\mathrm{Ca}}^{2+},K^{+}$), M is an element whose surroundings have the shape of a distorted octahedron (e.g., Mn, Fe, Li, Mg), and T is an element with a tetrahedral environment (e.g., P, As). The numbers in parentheses refer to different atomic positions [3]. The alluaudite structure consists of chains of M(2) octahedron pairs connected to each other by means of distorted M(1) octahedrons. The chains are connected through tetrahedra ${\mathrm{TO}}_{4}$ (e.g., ${\mathrm{PO}}_{4}$). As a result, they create surfaces extending in the plane (*a,c*). The three-dimensional structure of the alluaudite is the result of connecting these surfaces by tetrahedra ${\mathrm{PO}}_{4}$. This results in the formation of two types of channels in the direction *c* [3,4]. The channels may be filled with atoms X. There is more than one possible atom position in each channel. According to the nomenclature used in Hatert’s work, these will be A(1), A(1)’, and A(1)” in Channel 1 and A(2) and A(2)’ in Channel 2, respectively. Unlike the M positions, the channel positions may be partially or completely empty. In most alluaudites, the atoms are at positions A(1) and A(2)’, and there are gaps at positions A(1)’, A(1)”, and A(2) [5].

Crystalline compounds with the alluaudite structure can be also synthesized. Hatert in his work [5] described the synthesis of a compound ${\mathrm{NaMnFe}}_{2}{\left({\mathrm{PO}}_{4}\right)}_{3}$—with a similar chemical composition to the natural alluaudite originating from Buranga in Rwanda, studied earlier by Fisher. Synthesis by the solid-state reaction method resulted in obtaining a material with unit cell parameters compatible with the natural mineral [5]. The presence of channels in which unoccupied ion sites may additionally exist has attracted interest in these materials for use in reversible batteries. In 2003, Richardson presented preliminary electrochemical results, using ${\mathrm{NaFe}}_{3}{\left({\mathrm{PO}}_{4}\right)}_{3}$ as a cathode material in lithium ion batteries [6]. Usage of ${\mathrm{NaMnFe}}_{2}{\left({\mathrm{PO}}_{4}\right)}_{3}$ as a cathode in sodium batteries was demonstrated by Trad in 2010 [4].

A growing number of alluaudites with other stoichiometries and different transition metals have been reported, including iron, manganese, vanadium, and cobalt. Many of them were investigated as cathodes in sodium batteries, and their electrochemical capacity was determined. The values varied from 50 to 140 mAh/g, while most of the papers reported values about 100 mAh/g. A review of such parameters can be found, e.g., in [7]. Among the compositions containing iron, vanadium, and manganese, e.g., Na${}_{2}$Fe${}_{2}$V(PO${}_{4}$)${}_{3}$ [8] and Na${}_{2}$Mn${}_{2}$V(PO${}_{4}$)${}_{3}$ [9] have been synthesized and studied.

Redox reactions at the cathode side are limited by low ${\mathrm{Na}}^{+}$ ion diffusivity [10]. Low electrical conductivity is another obstacle for alluaudites studied to date for use in sodium batteries. The attempts to improve these drawbacks include coating the grains of the material with carbon, nanostructuring, filling the M position with various cations, or a combination of the above. Often, these treatments are insufficient, and it is necessary to add conductive carbon also at the stage of cathode preparation (e.g., [8,11,12,13]). However, this increases its volume and decreases the volumetric capacity. Therefore, it is worth trying to reduce its amount in electrochemical devices.

In recent years, J.E. Garbarczyk, T.K. Pietrzak, and coworkers investigated the phenomenon of the thermal nanocrystallization of amorphous analogues of cathode materials for lithium batteries (e.g., [14,15]). This method consists of two stages: (i) rapid cooling of the molten mixture of substrates and (ii) proper heat treatment of the glass in order to obtain nanocrystalline glass-ceramics. The advantage of this approach is the lack of carbon additives during the process. Nonetheless, as a result, nanomaterials often exhibited a giant increase of electrical conductivity, when containing crystalline grains of sizes up to several nanometers. The success of the method was attributed to the creation of favorable conditions for the electron (polaron) hopping conductivity on the surfaces of the nanograins.

A similar approach was applied recently to NASICON [16] and alluaudite-type [17] materials. It was shown that it is possible to obtain glassy analogues of these materials and subject them to thermal nanocrystallization. However, the observed increase of the conductivity was not very high. Furthermore, our in-depth studies revealed that the reproducibility of phase purity was low. Therefore, in this work, we decided to focus on optimizing the synthesis conditions to strive for the better phase purity and reproducibility, as well as higher conductivity of alluaudite-type nanostructured glass-ceramics. We selected the compositions reported previously in our paper [17], namely: Na${}_{2}$Fe${}_{3}$(PO${}_{4}$)${}_{3}$, Na${}_{2}$VFe${}_{2}$(PO${}_{4}$)${}_{3}$, and Na${}_{2}$VFeMn(PO${}_{4}$)${}_{3}$. According to our best knowledge, the latter composition has not been reported elsewhere.

## 2. Materials and Methods

For the syntheses, the following reagents were used: Na${}_{2}$CO${}_{3}$ (99.8%, Polish Chemicals), FePO${}_{4}\cdot 2$H${}_{2}$O (pure, Roth) or FeC${}_{2}$O${}_{4}\cdot 2$H${}_{2}$O (99%, Aldrich), V${}_{2}$O${}_{5}$ (99.6%, Roth), Mn(CH${}_{3}$COO)${}_{2}\cdot 4$ H${}_{2}$O (99%, Aldrich), NH${}_{4}$H${}_{2}$PO${}_{4}$ (99%, Polish Chemicals). Stoichiometric amounts of the reagents were homogenized in a mortar and put into porcelain crucibles. The crucibles were put in an inductive furnace (Argenta AFI–02) preheated to 700 °C and heated up to 1300–1350 °C, depending on the sample. The heating was carried out at the full power of the furnace (4 kW). This resulted in the heating rate reaching up to 100 °C/min. To ensure a nonoxidizing atmosphere, a double-crucible technique was used. In other words, the main crucibles were put into a larger crucible filled with activated charcoal and covered with a lid. The visualization of this concept can be seen in Figure 1 in [18]. The batches were kept at the maximum temperature for 15 min and eventually quenched between two metal plates (stainless steel or copper) in air.

The amorphousness of the as-synthesized samples and the structure of glass-ceramics were investigated with X-ray diffractometry. A Malvern Panalytical Empyrean diffractometer was used, equipped with a copper lamp. The measurements were carried out in the 5–110° 2θ range at room temperature. The temperatures of glass transition and crystallization were determined by differential thermal analysis. The measurements were carried out with a TA SDT Q600 device with a constant heating rate of 1 °C/min and 10 °C/min in a flow of argon. The temperature dependencies of the electrical conductivity were investigated with impedance spectroscopy using either Solartron 1260 or Novocontrol Alpha-A analyzers, in a tube furnace (manufacturer: *Czylok*). The measurements were carried out in an argon atmosphere. High temperature stabilization was ensured by a Eurotherm 2404 controller and self-developed software, as described in [19]. Due to the modest conductivity of the pristine glasses, a signal of 1 V amplitude was used in these experiments. The temperature was stabilized for at least 30 min prior to each measurement. The temperature drift during each impedance measurement was lower than 0.5 °C. The microstructure of the samples was observed using field emission scanning electron microscopy (FE-SEM) on a Zeiss Ultra Plus instrument at the Institute of High Pressure Physics, PAS. The cross-sections of the samples were sputtered with a thin (several nm) conducting layer of carbon. EDS elemental analyses of the glasses were performed during the SEM experiments.

## 3. Optimization of the Synthesis Route

The phase purity of alluaudite-type glass-ceramics synthesized previously by us [17] was still unsatisfactory and appeared to be partially irreproducible. Therefore, the most important part of this study was to adjust different parameters of the syntheses to obtain alluaudite-type nanostructured glass-ceramics with high phase purity. The following parameters were altered during subsequent syntheses of alluaudite-like glasses:

1. The time of melting;
2. The temperature of melting;
3. The source of iron (iron phosphate or iron oxalate);
4. Additional presynthesis before high-temperature melting;
5. Plates being used for quenching;
6. The use of an additional lid, providing a better reduction of the reagents;
7. The time between the homogenization of the reagents and the synthesis;
8. The amount of the reagents in the crucibles.

**Figure 1:**
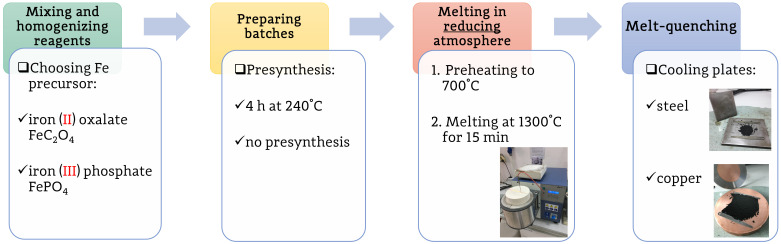
Scheme of the key factors taken into account during the optimization of the syntheses.

Due to numerous synthesis parameters (the key factors are presented in Figure 1), let us introduce the following system of sample identification: MMM-Fe-pp-ld-mq, where the meaning of the symbols are as follows: MMM—the presence of transition metals in the nominal composition, Fe—the iron source, pp—presynthesis, ld—covering lid, mq—cooling plates used for melt-quenching. The possible values of each parameter are—for clarity—listed in Table 1.

Previous investigations (both reported in [17] and other studies of ours, unpublished) indicated that the optimal time and temperature of the syntheses were 15 min at 1300 °C. Therefore, all further syntheses were carried out with these fixed conditions. Precisely, the batches were put into the outer crucible preheated to 700 °C, rapidly heated to 1300 °C, and kept at that temperature for 15 min, before quenching.

**Figure 2:**
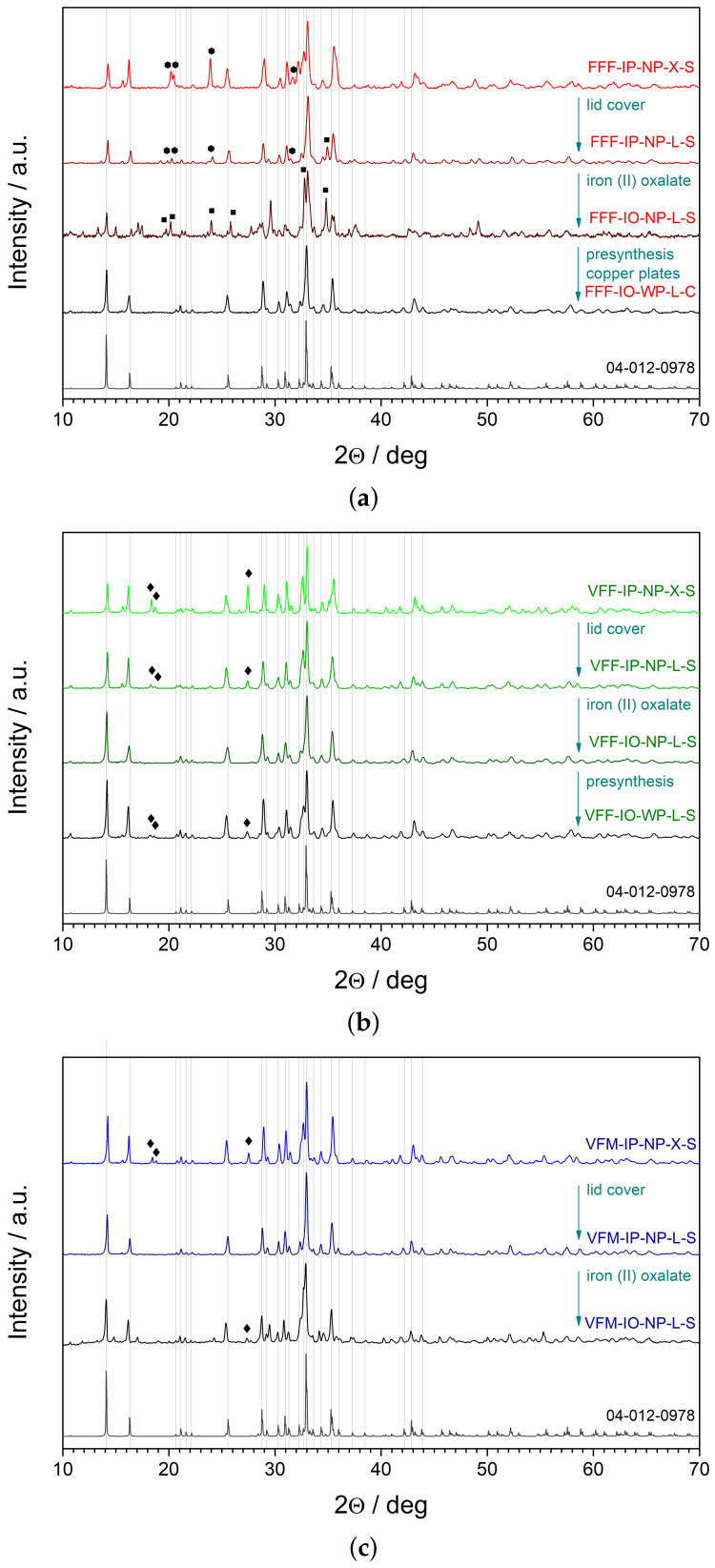
XRD patterns of glasses at various conditions, after their heat treatment at 650 °C: (**a**) Na${}_{2}$Fe${}_{3}$(PO${}_{4}$)${}_{3}$, (**b**) Na${}_{2}$VFe${}_{2}$(PO${}_{4}$)${}_{3}$, (**c**) Na${}_{2}$VFeMn(PO${}_{4}$)${}_{3}$. Gray vertical lines show the positions of the diffraction lines of the reference Na${}_{2}$Fe${}_{2}$Mn(PO${}_{4}$)${}_{3}$ alluaudite (ICDD Card No. 04-012-0978) pattern. Major peaks ascribed to impurity phases are marked as follows: hexagons—Na${}_{3}$Fe${}_{2}$(PO${}_{4}$)${}_{3}$ (ICDD Card No. 00-045-0319); squares—NaFePO${}_{4}$ (ICDD Card No. 01-083-9002); diamonds—NaVOPO${}_{4}$ (ICDD Card No. 04-009-5707).

The optimization started from samples FFF (i.e., with the nominal composition of Na${}_{2}$Fe${}_{3}$(PO${}_{4}$)${}_{3}$). In Figure 2, the XRD patterns of several synthesized samples after heat treatment at 650 °C are shown. Due to numerous syntheses with a wide variety of parameters, we reduced the data to show the most important factors affecting the final phase purity. We started from the synthesis using iron (III) phosphate as a precursor, no presynthesis, no additional cover, and quenching with stainless steel plates (FFF-IP-NP-X-S). These parameters resulted in a sample with the alluaudite phase with considerable Na${}_{3}$Fe${}_{2}$(PO${}_{4}$)${}_{3}$ secondary phase impurities (ICDD Card No. 00-045-0319). In Na${}_{3}$Fe${}_{2}$(PO${}_{4}$)${}_{3}$, iron exists at the 3+ oxidation level, whereas in alluaudite, the average oxidation level is 7+/3. Therefore, we decided to cover the outer crucible with an additional lid (FFF-IP-NP-L-S), providing a better reducing atmosphere, which resulted in a much lower content of the secondary phase. However, traces of orthorhombic NaFePO${}_{4}$ (ICDD Card No. 01-083-9002) were detected. We also tried to substitute iron (III) phosphate with iron (II) oxalate (FFF-IO-NP-L-S). At first, the results were disappointing. The batch would not easily glassify, and further heat treatment resulted in significant amounts of NaFePO${}_{4}$ and other, unidentified phase impurities. The use of presynthesis was previously experienced as a factor improving phase purity. To further facilitate the glassification process, we changed the stainless steel plates to copper ones, which would provide a faster cooling. The batch synthesized in such conditions (FFF-IO-WP-L-C) resulted in superior phase purity after nanocrystallization.

One might expect that similar conditions would be also optimal for the syntheses of the samples VFF (i.e., with the nominal composition of Na${}_{2}$VFe${}_{2}$(PO${}_{4}$)${}_{3}$). Unfortunately, this was not the case, and therefore, various parameters were checked once again. We started from syntheses using iron (III) phosphate, no presynthesis, and no additional lid (VFF-IP-NP-X-S). This resulted in samples with NaVOPO${}_{4}$ impurities (ICDD Card No. 04-009-5707). In NaVOPO${}_{4}$, vanadium exists at the 4+ oxidation level, whereas in alluaudite, its oxidation state should be 3+. Therefore, a more reducing atmosphere was provided with the use of an additional lid (VFF-IP-NP-L-S). This resulted in reduced amounts of the NaVOPO${}_{4}$ secondary phase. Substitution of the iron source (with iron oxalate) resulted in a material with high phase purity (VFF-IO-NP-L-S). The influence of the presynthesis was additionally checked (VFF-IO-WP-L-S). Surprisingly, in this case, the procedure reduced the phase purity by introducing the NaVOPO${}_{4}$ secondary phase.

The beginnings of VFM optimization (i.e., Na${}_{2}$VFeMn(PO${}_{4}$)${}_{3}$) started from the synthesis using iron phosphate without the presynthesis (VFM-IP-NP-X-S). This procedure resulted (after further heat treatment at 650 °C) in materials with the alluaudite structure containing NaVOPO${}_{4}$ impurities. In this case, however, the use of an additional lid (VFM-IP-NP-L-S) was enough to obtain the sample with the pure alluaudite structure. It was also checked whether the use of the presynthesis affected the final phase purity. In such a procedure (VFM-IP-WP-L-S, not shown in the figure), minor impurities of NaVOPO${}_{4}$ were detected. Substitution of iron phosphate with iron oxalate (VFM-IO-NP-L-S and VFM-IO-WP-L-S) also resulted in materials with poor phase purity. Additionally, the procedure without the presynthesis caused much lower glass-forming properties of the batch. It is worth mentioning that, sometimes, the procedure VFM-IP-NP-L-S was not fully reproducible, i.e., it resulted in minor phase impurities. This issue was carefully investigated. The period between the homogenization of the reagents and the synthesis appeared to play a noticeable role. The satisfactory phase purity was obtained when the reagents were kept for several days in an ambient atmosphere and standard humidity. There is no quantitative explanation of this phenomenon. However, in our opinion, it was the low humidity (e.g., caused by air conditioning) that had a negative influence on the final phase stability.

The purpose of these efforts was to obtain alluaudite nanocrystalline materials with high phase purity. Additionally, we observed the influence of the quenching rate on the glassification of the as-received materials. In particular, it was possible to obtain glassy FFF only by quenching between copper plates. Cooling with stainless steel plates (lower rate) usually resulted in partially crystallized materials. However, the primary phase was in agreement with alluaudite reference patterns. The phase persisted also after their thermal treatment. The compositions VFF and VFM did not require quenching with copper plates to obtain satisfactorily amorphous samples.

## 4. Results

The samples with the highest phase purity from the previous section were selected for further in-depth investigation, namely: FFF-IO-WP-L-C, VFF-IO-NP-L-S, and VFM-IP-NP-L-S. In the following text and figures, they are referred to simply as FFF, VFF, and VFM, respectively.

### 4.1. Differential Thermal Analysis

**Figure 3:**
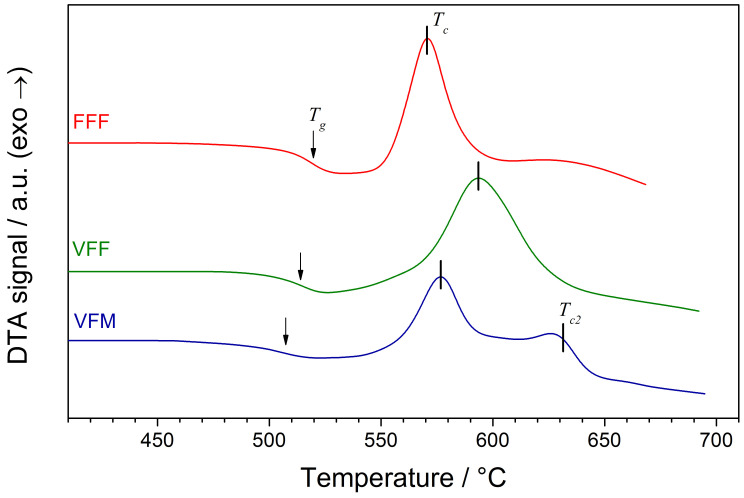
DTA traces of as-synthesized glasses. The measurements were carried out with a 10 °C/min heating rate in argon flow. The glass transition and crystallization processes are marked with arrows and vertical lines, respectively.

Differential Thermal Analysis (DTA) traces of the as-synthesized glasses are shown in Figure 3. Their shape is typical for glassy materials. An endothermic step-like glass transition was followed by a distinct exothermic crystallization process. The temperatures of the observed thermal events (namely glass transition $T_{g}$ and main crystallization $T_{c}$) are shown in Table 2. In the sample VFM, an additional crystallization process was observed at a higher temperature (peak maximum at $T_{c2}=623$ °C).

### 4.2. X-ray Diffractometry

**Figure 4:**
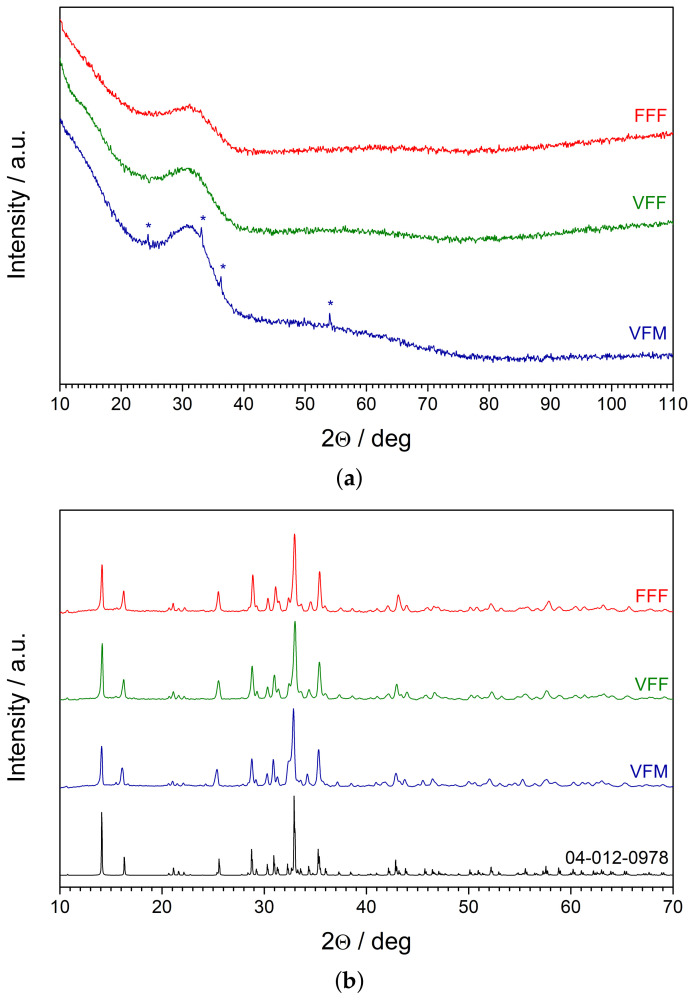
XRD patterns of the as-synthesized glasses (**a**) and samples after heat treatment at 650 °C (**b**). The impurities of the V${}_{2}$O${}_{3}$ karelianite phase (ICSD Card No. 98-000-1871) are marked with the asterisks. A pattern of Na${}_{2}$Fe${}_{2}$Mn(PO${}_{4}$)${}_{3}$ alluaudite (ICDD Card No. 04-012-0978) is given as a reference for the nanocrystalline samples.

The XRD patterns of the as-synthesized glasses are shown in Figure 4a. All samples exhibited an amorphous halo and lacked significant Bragg reflexes, which confirmed their amorphousness. Only in the pattern of the samples of VFM, several minor peaks were observed. They were ascribed to the V${}_{2}$O${}_{3}$ karelianite phase (ICSD Card No. 98-000-1871). This proved that the V${}_{2}$O${}_{5}$ used as a reagent in the synthesis was reduced to vanadium (III) oxide, but was not incorporated entirely into the glass network.

The patterns of glassy samples that were subject to heat treatment at 650 °C are shown in Figure 4b. A pattern of Na${}_{2}$Fe${}_{2}$Mn(PO${}_{4}$)${}_{3}$ alluaudite [20] (ICDD Card No. 04-012-0978) is given as a reference. Two conclusions can be drawn from this plot: (i) all nanocrystallized samples (containing different transition metals) preserved the monoclinic structure of alluaudite; (ii) no impurity phases were detected besides the primary alluaudite phase. Furthermore, the precipitates of V${}_{2}$O${}_{3}$ that were observed in the glassy VFM samples were no longer detected by XRD. This is probably because the V${}^{3+}$ ions were incorporated into the alluaudite structure.

### 4.3. Impedance Spectroscopy

In general, the values of the electrical conductivity of the as-synthesized glasses were, as expected, modest and slightly varied among the samples from the same batch. Therefore, several measurements were carried out for different fragments, and average values were calculated. This procedure yielded conductivity ($\sigma_{g}$) as follows: $1.5\left(2\right)\times 10^{-8}$ S/cm, $2\left(1\right)\times 10^{-10}$ S/cm, and $3.5\left(9\right)\times 10^{-11}$ S/cm for batches FFF, VFF, and VFM, respectively.

**Figure 5:**
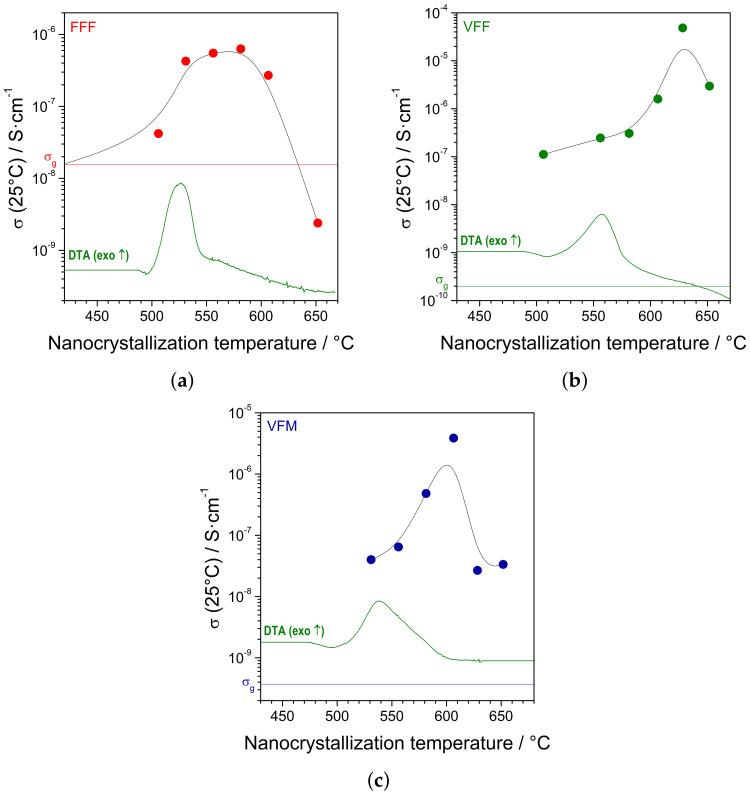
Electrical conductivity at room temperature of samples after their nanocrystallization at different maximum temperatures: (**a**) FFF, (**b**) VFF, (**c**) FVM. The conductivity of the starting glass ($\sigma_{g}$) is marked with a horizontal line. DTA traces taken at 1 °C/min are given for comparison.

Subsequently, the glassy samples were heat-treated in argon flow up to different maximum temperatures (with a heating rate of 2 °C/min) and cooled down to room temperature. The electrical conductivity of each nanocrystallized sample was measured. The summary results are presented in Figure 5a–c. It was shown that the proper heat treatment led to a significant increase in the conductivity. The increase was significantly different depending on the composition. The conductivity of nanocrystallized FFF samples was only ca. 100-times higher than the starting glass. On the contrary, the conductivity of nanocrystallized VFF and VFM samples was approximately five orders of magnitude higher.

Impedance spectroscopy carried out in situ during the heating and subsequent cooling ramps gave a better insight into the influence of the nanocrystallization on the electrical conductivity in the samples. When planning these experiments, the maximum temperature was selected accordingly to the highest conductivity reached previously in ex situ measurements.

From these measurements, the values of activation energy ($E_{a}$) of conductivity were determined: 0.57 eV, 0.71 eV, and 0.88 eV for the FFF, VFF, and VFM glasses, respectively. After nanocrystallization, $E_{a}$ slightly dropped in the case of FFF to 0.46 eV. For other compositions, the decrease was more pronounced, i.e., 0.21 eV and 0.11 eV for the VFF and VFM nanomaterials, respectively.

**Figure 6:**
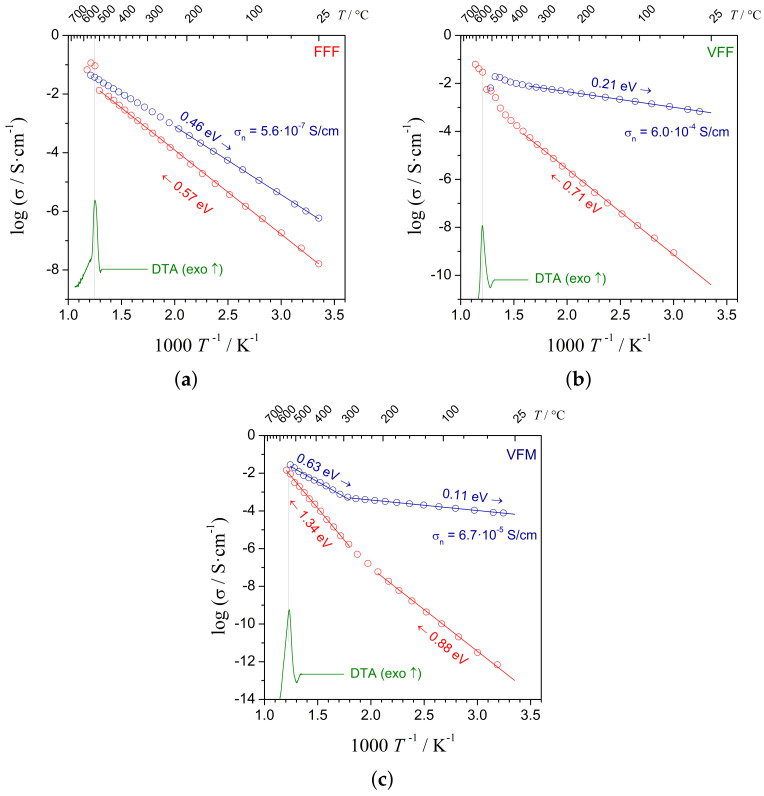
Temperature dependencies of electrical conductivity upon heating and subsequent cooling of initially glassy samples: (**a**) FFF, (**b**) VFF, (**c**) VFM The values of the activation energy upon heating and cooling are given, along with the value of the room temperature conductivity of the nanomaterials. Corresponding DTA traces taken at 1 °C/min are provided for comparison. The estimated uncertainties of the values are smaller than the least significant digit in the notation.

Analyses of the shape of the impedance figures revealed that both glasses and highly conductive nanomaterials exhibited predominant electronic conductivity. The Nyquist plots showed well-defined single semicircles. Only in the case of the VFM samples, high-temperature impedance spectra showed low-frequency spurs originating from ionic conductivity. This is the reason why the VFM samples exhibited different (higher) activation energies (Figure 6c) in a high-temperature range.

### 4.4. Scanning Electron Microscopy with EDS Microanalysis

During SEM imaging, the average elemental composition of the as-synthesized glasses was studied by EDS. The main purpose was to identify impurities (i.e., the elements not present in the nominal compositions) and their content. It came as no surprise that minute amounts of aluminum and silicon were detected. The ceramic crucibles used in the syntheses were said to be the origin of these elements. Their content was, however, negligible and varied between 0.2–0.4 at.% and 0.4–0.7 at.% for aluminum and silicon, respectively.

**Figure 7:**
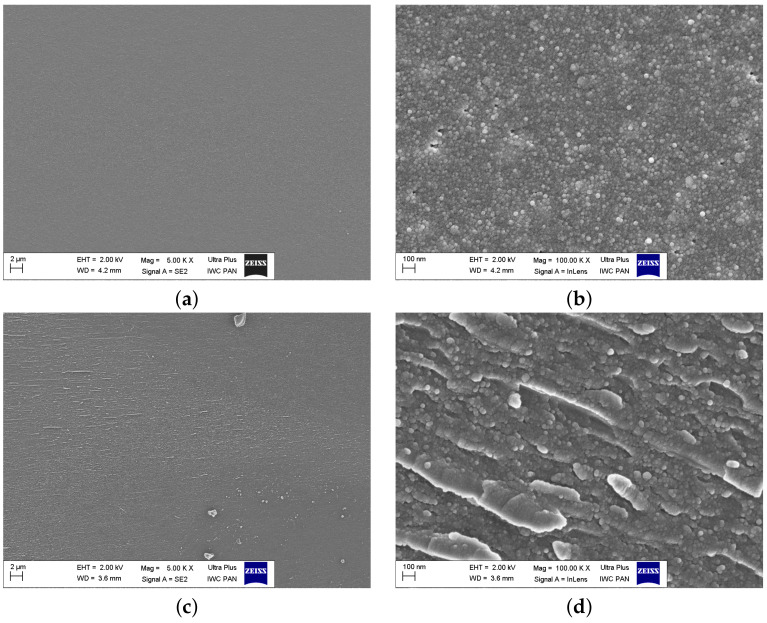

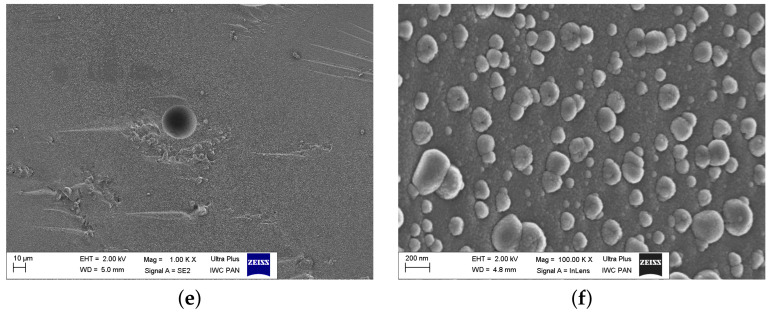
SEM images of the as-synthesized glassy samples taken at low magnifications (1–5 kX, left images) and high magnifications (100 kX, right images): FFF (**a**,**b**), VFF (**c**,**d**), VFM (**e**,**f**).

SEM images of the as-synthesized glasses (Figure 7a,c,e) taken at low magnifications showed, in general, a uniform amorphous structure without noticeable polycrystalline grains. However, high magnifications revealed the microstructure of the samples consisting of fine grains. In the sample FFF, the dimensions of the grains were ca. 10 nm. The sample VFF exhibited indistinct structures aligned in one direction. This alignment was more clearly visible at higher magnification (Figure 7d). This was probably the result of the melt-quenching method, where the orientation was determined by the cooling plates. The microstructure of the VFM sample exhibited some other inhomogeneities, e.g., pores and infrequent grains. An image taken at high magnification (Figure 7f) revealed ca. 100 nm grains precipitated in the surrounding matrix. The origin of these grains was unclear. These might have been SiO${}_{2}$ gains, but SEM images in the backscattered mode showed no distinct contrast between the matrix and the grains. This suggested that there was no significant difference in composition (precisely: the average atomic number of the elements) between these features. Furthermore, the XRD measurements of all samples visible in Figure 7a–f showed no Braggs’ reflexes. Hence, we came to the conclusion that the nanograins were either amorphous or were the nuclei of crystalline grains that would grow after thermal treatment. This would be in agreement with the facts that it was not easy to synthesize entirely amorphous samples and we were on the very edge of glassification-crystallization region.

**Figure 8:**
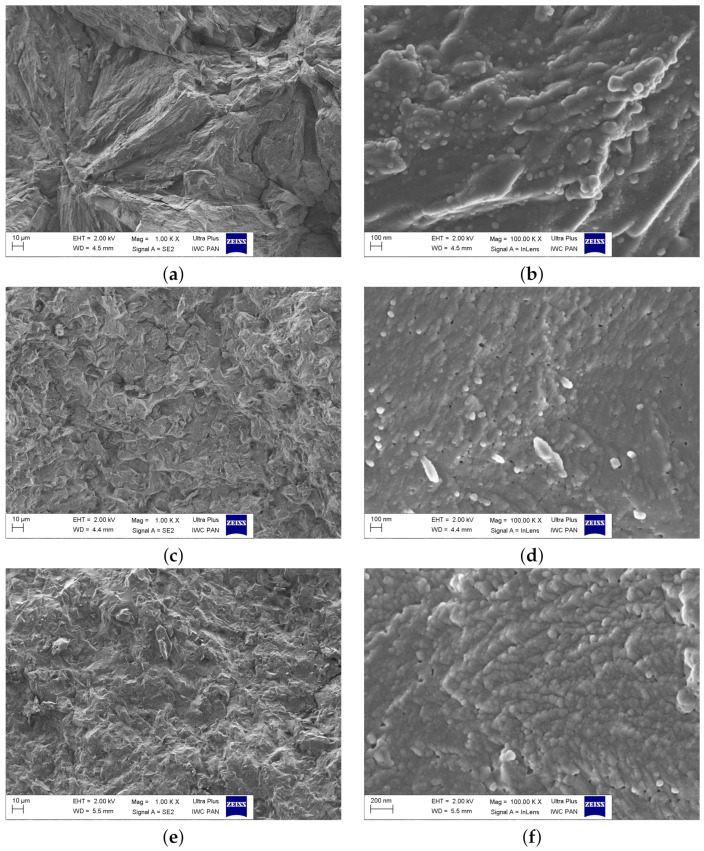
SEM images of the nanocrystallized samples with significantly improved electrical conductivity, taken at low magnifications (1 kX, left images) and high magnifications (100 kX, right images): FFF (**a**,**b**), VFF (**c**,**d**), VFM (**e**,**f**).

The SEM images of the samples after thermal treatment are shown in Figure 8a–f. At first, at low magnifications, their microstructure resembled polycrystalline materials, as large (micrometric) structures were visible. However, at higher magnifications, a more complex microstructure was revealed. The large grains were composed of smaller nanometric grains, which were mostly pronounced in the sample VFM (Figure 8f). As was demonstrated earlier (e.g., [14,15]), the presence of nanocrystallites with disordered shells provides favorable conditions for electronic transport due to polaron hopping between aliovalent transition metal ions.

## 5. Discussion

The studies of the alluaudite-type glassy and nanostructured materials presented in this paper were in agreement with other reports on this phenomenon in various glass systems. At the same time, some differences between this study and our previous work about alluaudites [17] need to be underlined.

DTA traces in this work (Figure 3) and in the previous work (Figure 1 in [17]) slightly differed. The glass transition and crystallization peaks were now more distinct. Previously, DTA traces contained additional minor peaks, probably resulting from the crystallization of secondary phases. Now, only VFM glass exhibited two crystallization peaks. This was, however, not reflected in the XRD patterns, where only the primary alluaudite phase was observed. One can conclude that the secondary peak may originate from the recrystallization processes of the primary phase.

The temperatures of the thermal events varied between compositions. Such an effect was observed earlier many times in different glass systems, e.g., LiF–M${}_{2}$O${}_{3}$–P${}_{2}$O${}_{5}$ [21] or NaF–M${}_{2}$O${}_{3}$–P${}_{2}$O${}_{5}$ [16]. Different transition metals create bonds of various strengths and therefore affect the temperatures of the glass transition and crystallization. In addition to this, the synthesis conditions may have an influence on the temperatures of thermal events occurring in samples with the same compositions. Such an effect was observed, e.g., in vanadate–phosphate glasses [22].

The alluaudite-type cathode materials studied in this work belong to a class of mixed electronic ionic conductors. The ionic part is due to mobile Na${}^{+}$ ions. Electronic conduction occurs due to polaron hopping between transition metals at various oxidation levels, e.g., Fe${}^{2+}$/Fe${}^{3+}$ or V${}^{3+}$/V${}^{4+}$ [15]. In this work, higher values of electrical conductivity than previously found were observed. The conductivity of the initial glasses ranged roughly between $10^{-10}$ and $10^{-8}$ S/cm. This was probably because more favorable conditions for electron hopping appeared. In the previous work [17], the conductivity of nanocrystalline alluaudites was still modest (mostly between $10^{-12}$ and $10^{-11}$). In this work, as a result of systematic studies, nanostructured materials with significantly improved conductivity were obtained. Previously, the nanocrystallized samples exhibited predominant ionic conductivity. Now, the electronic part was significantly improved. Thus, the samples exhibited predominant electronic conductivity. The decrease of the activation energy after nanocrystallization is a typical phenomenon. Values as low as 0.1–0.2 eV were observed, e.g., in highly conducting olivine-like nanomaterials [14]. In Figure 5, one can observe the changes of the conductivity of the nanomaterials at room temperature depending on the nanocrystallization (i.e., heat treatment) maximum temperature. The increase began around a crystallization peak observed in the DTA trace and was the highest soon after the peak. Further heating resulted in a conductivity drop. A similar behavior was observed previously in vanadate–phosphate glasses (Figure 14 in [23]).

One can see that the highest values of the conductivity presented in Figure 5 slightly differed from the values obtained during in situ measurements (Figure 6). This was probably because the final value of conductivity was very sensitive to the nanocrystallization temperature. For both heat treatments, furnaces of the same model were used. However, in the case of in situ measurements, the sample was held in a holder in the middle of the tube. In the other case, the samples were put in alumina combustion boats lying at the bottom of the tube. Thus, even though the set temperature of the furnace was the same, the different geometry could make a noticeable difference in the exact temperature of the heat-treated samples.

The values of the conductivity of the nanocrystallized samples were in qualitative agreement with their microstructure. A lower conductivity was observed in the FFF sample. In this material, the amount of small nanocrystallites (Figure 8b) was lower than in other materials. VFF and VFM exhibited higher conductivity, and their microstructure was dominated by nanocrystallites (Figure 8d,f). Such a correlation was previously observed, e.g., in the case of olivine-like nanomaterials [14].

## 6. Conclusions

In this work, a systematic optimization of the synthesis conditions of three alluaudite-like glasses was conducted: Na${}_{2}$Fe${}_{3}$(PO${}_{4}$)${}_{3}$ (FFF), Na${}_{2}$VFe${}_{2}$(PO${}_{4}$)${}_{3}$ (VFF), and Na${}_{2}$VFeMn(PO${}_{4}$)${}_{3}$ (VFM). Numerous factors that had an influence on glassification and the final phase purity of the nanomaterials were identified. They were, however, not the same in all cases. The optimal synthesis conditions for the samples FFF were as follows: use of iron oxalate, presynthesis, melt-quenching with copper plates. In the case of VFF, the conditions were: use of iron oxalate, no presynthesis, melt-quenching with steel plates. In the latter case (VFM), iron phosphate was used, no presynthesis, and melt-quenching with steel plates. In all cases, the additional covering with a lid was beneficial for the phase purity.

The selected samples were subsequently subject to a systematic optimization of the nanocrystallization conditions. This resulted in samples with a microstructure consisting of small nanocrystallites and much improved conductivity. The highest value was observed for the VFF composition and was equal to $6\times 10^{-4}$ S/cm. The electrical conductivity of the other nanocrystalline samples reached $5.6\times 10^{-7}$ S/cm (FFF) and $6.7\times 10^{-5}$ S/cm (VFM). In all cases, the conductivity exhibited a predominant electronic nature. In these mixed conductors, the ionic conduction must have been lower by at least an order of magnitude and therefore not observed in the impedance spectroscopy measurements. This is, however, also important for electrochemical performance in NIBs. Therefore, the electrochemical properties of these materials will be studied in the near future.

## Figures and Tables

**Table 1:** Key to the symbols used in the sample identification system MMM-Fe-pp-ld-mq.

Category	Value	Meaning
MMM	FFF	Na${}_{2}$Fe${}_{3}$(PO${}_{4}$)${}_{3}$
(nominal composition)	VFF	Na${}_{2}$VFe${}_{2}$(PO${}_{4}$)${}_{3}$
	VFM	Na${}_{2}$VFeMn(PO${}_{4}$)${}_{3}$
Fe	IO	iron oxalate
(iron source)	IP	iron phosphate
pp	WP	with presynthesis
(presynthesis)	NP	no presynthesis
ld	L	crucible covered with a lid
(cover)	X	crucible not covered with a lid
mq	S	stainless steel plates
(cooling plates)	C	copper plates

**Table 2:** Glass transition ($T_{g}$) and crystallization ($T_{c}$) temperatures in the studied glassy samples, determined from DTA measurements carried out with a 10 °C/min heating rate in argon flow.

Sample ID	$T_{g}$/°C	$T_{c}$/°C
FFF	519.3	570.5
VFF	514.9	593.7
VFM	507.3	576.1

## Data Availability

Data sharing not applicable.

## Abbreviations

The following abbreviations are used in this manuscript:

XRD	X-ray Diffractometry
DTA	Differential Thermal Analysis
IS	Impedance Spectroscopy
SEM	Scanning Electron Microscopy
EDS	Energy Dispersive Spectroscopy
